# Sport Experience and Physical Activity: Event-Related Brain Potential and Task Performance Indices of Attention in Young Adults

**DOI:** 10.3390/jfmk4020033

**Published:** 2019-06-04

**Authors:** Mohamed Aly, Mohamed A. Ahmed, Asmaa Hasan, Haruyuki Kojima, Abdelhakem R. Abdelhakem

**Affiliations:** 1 Institute of Human and Social Sciences, Department of Psychology, Kanazawa University, Kanazawa 920-1192, Japan; 2 Faculty of Medicine, Department of Neurology and Psychiatry, Assiut University, Assiut 71515, Egypt; 3 Faculty of Medicine, Department of Neurology and Psychiatry, Assiut University, Assiut 71515, Egypt; 4 Faculty of Physical Education, Department of Physical Psychological and Education Sciences, Assiut University, Assiut 71515, Egypt

**Keywords:** physical activity, swimmers, karateka, ERP, P3

## Abstract

A growing body of literature demonstrates that engaging in sport regularly and maintaining an active lifestyle have a positive impact on cognition. The purpose of this study was to examine the influence of sport experiences and physical activity on attention, and explore whether the type of sport can impact differently on the neuroelectric system using Event-Related Potentials (ERPs). Thirty-three young adults (mean age = 19.72 ± 1.25) were divided according to their sport experience into swimmers, karateka, and irregular exercisers. Participants performed auditory oddball tasks, while measures of task performance and ERPs were collected. The results indicated that exercisers, regardless of their sport experience, exhibited a larger and shorter P3 compared to irregular exercisers. However, no significant difference was observed in the reaction time (RT) between groups. No statistically significant differences in the RT and P3 were present between swimmers and karateka. These findings suggest that sport experiences, regardless of the type, are associated with a larger amount of neural attentional resources and faster stimulus evaluation speed. The results replicate previous studies that have reported improved cognitive functions in more active individuals. They further extended the current knowledge by indicating that both swimming and karate influence attention and do not differentially alter the brain response.

## 1. Introduction

The effects of motor experience and physical activity on cognition have received growing attention in the exercise–cognition domain. With the increase of scientific evidence, studies have demonstrated that exercise may not only ameliorate cognitive decline and impairment, but can also achieve cognitive enhancement across the lifespan [1,2,3].

The beneficial relation of chronic and acute participation in physical activity to brain health and cognition arises from the crucial role of physical activity in increasing the growth factor chemicals, improving the abundance and survival of new brain cells, and creating strong functional connectivity [4,5,6]. These neurological and physiological changes are evident when comparing high-activity and low-activity people [7,8]. A meta-analysis by Ludyga (2016) found that in 40 empirical studies published between 2005 and 2015, exercise induced benefits on specific aspects of executive function in different age and aerobic fitness subgroups [9]. In a review paper on the relation of chronic participation in physical activity on aspects of cognition, the data support a beneficial relationship between long-term participation in physical activity and brain health and cognition. These data indicate that physical activity may influence brain health and cognition, leading to better overall effective functioning [10]. Based on a review of the extant literature, regular engagement in exercise can provide a simple means for healthy people to optimize a range of executive functions [3], and appears as a noninvasive and effective strategy for counteracting neurological and cognitive disorders [3,11].

In exercise–cognition research, behavioral measures are essential in evaluating cognitive functions. However, Event-Related Potentials (ERPs) have provided additional insight into the underlying mechanisms that occur during cognitive processing. The high temporal resolution (i.e., milliseconds) of the ERP technique allows researchers to measure a subset of covert cognitive processes and provides information regarding the mechanisms underlying cognitive functioning above and beyond the overt behavior [12]. ERP components are described by a set of positive (P) and negative (N) deflections, which are composed according to their direction and the relative time in which they occur [13].

Although several ERP components have been used to study the relationship between physical activity and cognitive functions, P300 (also known as P3 or P3b) has often been employed in this area of research. P3 is a positive component occurring approximately 300–800 ms after stimulus representation [14]. The timing of P3 component occurrence changes depending on the given task and subjects’ age. The P3 component is considered to be the signature of cognitive processes such as attention and working memory. Furthermore, P3 is widely used for examining aspects of information processing related to stimulus engagement. In other words, P3 reflects the allocation of attentional resources associated with working memory processes during context changing of the stimulus environment once sensory information has been analyzed [15]. The amplitude of P3 is thought to reflect the neural representation of the stimulus environment. Thus, increasing P3 amplitude indicates an increase in the attentional resources allocated to the given stimulus [16]. P3 latency represents the cognitive processing speed, so the longer P3 latency is associated with a delay in information processing time [14,17]. P3 physical activity studies have indicated that higher levels of physical activity are associated with larger P3 amplitude and/or short P3 latency during stimulus discrimination tasks [18,19,20,21,22]. 

The classification of open-skill and closed-skill sports depends on the predictability of performing the environment during sports practices [23]. Open-skill sports require individuals to invest more cognitive effort in response to immediate external stimuli that arise from an unpredictable environment. A few recent studies investigated whether a specific cognitive change could be associated with a particular type of exercise or sport. Dai et al. (2013) examined how exercise type can influence P3 and task-switching performance among older adults who engaged in open-skill activities (e.g., football and tennis), closed-skill activities (e.g., swimming and jogging), and low-activity lifestyle. Task switching paradigm generally examines the ability to shift attention from one task to another. The difference in the reaction time (RT) between the non-switch condition and switch condition is referred to as switching cost [24]. Dai et al. reported that open-skill and closed-skill exercisers had shorter RT and larger P3 amplitude relative to the irregular physically active counterparts [25]. Although the results revealed no differences between open-skill and closed-skill groups either in RT or P3, open-skill exercisers exhibited smaller switching cost compare to closed-skill which could be interpreted as open-skill sports may have more efficiency in cognitive flexibility [26,27] and working memory [24,28] than closed-skill sports. Thus, more cognitive demands in open skill sports may provide additional facilitation for cognition. 

The notion that higher cognitive demand during open-skill sports can provide additional facilitation for cognition was supported by Matthews and Williams (2008) and Taylor-Piliae et al. (2010). They suggested that Tai Chi Chouan could be beneficial for executive function because of its exercise modality that requires concentrating on body postures and movement sequences while exercising physically [29,30]. Other studies reported similar results with experienced fencers, indicating that fencer experience is associated with more rapid simple and discriminative RTs [31] and better inhibitory control [32] than those with no fencing experience. Taken together, a greater amount of cognitive investment in physical exercise can demonstrate more promising benefits to cognitive function when compared to physical exercise that involves a relatively smaller cognitive requirement. However, we should mention that the previous studies mainly focused on fencing as an open-skill sport, and there is a lack of studies that examined the cognitive effect of open-skill and closed-skill sports. Therefore, it is essential to investigate whether open-sports can offer additional cognitive benefits than closed-skill sports or not.

This study aimed to investigate the influence of sport experience and physical activity on the neuroelectric system. It was hypothesized that: (A) regular exercisers (swimmers, karateka), regardless of the type of sport, would demonstrate an increase in attention ability and faster stimulus classification speed; and (B) different sport backgrounds may cause different cognitive changes. In more instances, karateka (kumite), as an open-skill sport, may exhibit better performance compared to swimmers, as a closed-skill sport, in behavioral and/or neuroelectric levels.

## 2. Materials and Methods 

### 2.1. Participants

A cross-sectional design was employed in the present study. Thirty-three students aged 18–21 years were recruited from Assiut University. Participants were classified into one of three groups (swimmers, karateka, and irregular exercisers) based on their habitual sport participation and physical activity level. The swimmers and karateka groups included subjects currently engaged in karate or swimming training (at least five years of training, three times per week, and 60 min each per session). The irregular exercise group included the subjects who were not members of a sports club or had a regular exercise habit in their daily life routine. All participants were right-handed and had a normal or corrected-to-normal vision, and they reported being free of psychiatric, neurological, and motor disorders. 

### 2.2. Sport Experience and Physical Activity

Subjects underwent a sport experience survey designed by the authors for this investigation to classify the subjects according to their sport experiences. The short version of the International Physical Activity Questionnaire (IPAQ) [33] is an 8-item scale that estimates the amount of time spent in physical activity through three different intensities (vigorous, moderate, and light) and the general sedentary behavior during the previous seven days. The amount of Metabolic Equivalent (MET) minutes was calculated by multiplying the minutes per week of activity by 8 METs (vigorous), 4 METs (moderate), or 3.3 METs (walking) [34]. The total amount of physical activity was calculated by adding the MET-minutes for all three physical activity intensities and is further classified into three activity levels representing lifestyle routine using the following cut-off points: inactive person (<600 METs min/week), moderate-active person (600–2000 METs min/week), and high-active person (>2000 METs min/week) [35].

### 2.3. Auditory Oddball Task

Participants completed an auditory oddball task. The task required participants to press a button to infrequently presented target stimulus (20% probability), while no response was required for the frequent non-target stimulus (80% probability). The stimuli were a series of binaural 1000 Hz (non-targets) versus 2000 Hz (target) tones at 70 dBHL with a 10 ms rise/fall and 40 ms plateau time. Tones were presented at a rate of 0.9/s with target tones occurring randomly with a 0.2 probability. Subjects were instructed to press a button with the right thumb as quickly and accurately as possible to the infrequently target stimulus and to ignore the frequent non-target stimulus.

### 2.4. Experimental Procedure

After arriving at the laboratory, each subject was informed of the requirements of the experiment. Next, the IPAQ and sport experience survey were completed. Electroencephalography (EEG) was measured on a separate day. The neuroelectric measurement was set up according to the standard procedure of the Society for Psychophysiological Research. After verifying the clarity of the electrophysiological signals, subjects were instructed about the rules of the task. The entire session lasted approximately 45 min for each subject. EEG measurements were conducted at the Laboratory of Neurology and Psychiatry, Assiut University Hospital, Assiut. Prior to the experiment, written consent was obtained from all subjects, and the study was approved by the Ethical Review Board of Assiut University (Ethics ID. PE-2017-024) on 15 June 2017.

### 2.5. Event-Related Potential Recording

Event-related potentials (ERPs) were elicited with the auditory oddball task. Evoked potentials were recorded from the scalp electrodes placed at Fz, Cz, and Pz and were referenced to linked ear. Filter settings were 0.5 and 70 Hz, analysis time 1000 ms (from 100 ms before to 900 ms after the stimulus onset). Two or three trials were performed to demonstrate the consistency of the waveforms. Latency and amplitude of P3 were measured from stimulus onset to the positive peaks with a range of 200–500 ms. EEG measurements were conducted using a Nihon Kohden equipment model (7102), Surpass EP software. 

### 2.6. Statistical Analysis

A one-way ANOVA was separately conducted to the age, the education level, the body mass index (BMI), the characteristic of sport training, the amount of physical activity, and RT among the three groups. P3 amplitude and latency were submitted to 3 (group) × 3 site (Fz, Cz, and Pz) ANOVA to examine the interactions of group and site in neuroelectric measures. Post-hoc comparisons were conducted using Turkey’s honestly significant difference procedure. The effect size of partial-eta2 (η2) was reported for significant effects, where the alpha levels were set at 0.05. Data analyses were performed using SPSS 21.0 software system.

## 3. Results

### 3.1. Participants Characteristics

Table 1 provides the subjects’ characteristics of the three groups. A comparison of the amount of physical activity between groups using a one-way ANOVA indicated that the swimmers (2205.45 ± 213.21 MET) and karateka (2148.18 ± 248.47 MET) performed a larger amount of physical activity compared to the irregular exercisers (456.36 ± 294.02 MET), (*F*_2, 32_ = 134.58, *p* < 0.01, *η*^2^ = 0.90), which confirmed the regular exercise behavior groupings. Post-hoc comparison revealed no significant difference in physical activity between swimmers and karateka (*p* = 0.9). Group differences were not observed with respect to age, years of education, and BMI (*F*_2, 32_ = 0.74, *p* = 0.49, *η*^2^ = 0.05; *F*_2, 32_ = 0.49, *p* = 0.61, *η*^2^ = 0.04; *F*_2, 32_ = 2.53, *p* = 0.09, *η*^2^ = 0.15, respectively). These results revealed the homogeneity between the groups.

With regards to sport experience, there were no significant differences between swimmers and karateka in years of training, frequency, and duration of exercise (*p* > 0.05), which confirmed the parity between swimmers and karateka.

### 3.2. RT

RT analysis revealed that there was no significant effect among groups in response time (*F*_2, 32_ = 0.48, *p* = 0.62, *η*^2^ = 0.03).

### 3.3. ERP Analysis

#### 3.3.1. P3 Amplitude

**Figure 1:**
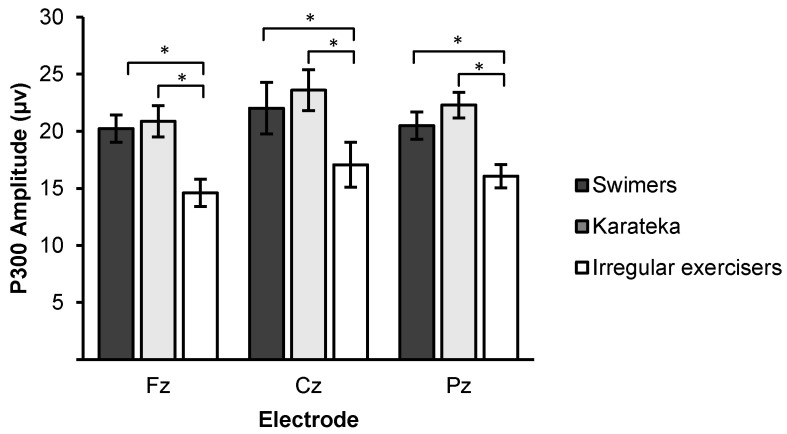
Mean P3 amplitude from the swimmers, karateka, and irregular exercisers during auditory oddball task. * significant difference at *p* > 0.05.

Two-way ANOVA of group (swimmers, karateka, and irregular exercisers) × site (Fz, Cz, and Pz) of P3 amplitude revealed a main effect of group (*F*_2, 32_ = 5.28, *p* = 0.011, *η*^2^ = 0.26), with larger P3 amplitude in the swimmers (20.92 ± 4.01 µV; *p* < 0.05) and karateka (22.26 ± 6.41 µV; *p* < 0.01) than the irregular exercisers (15.91 ± 3.60 µV), while there was no significant difference between the swimmers and karateka (*p* > 0.05). Two-way ANOVA of group × site revealed a main effect of site (*F*_2, 32_ = 6.37, *p* < 0.05, *η*^2^ = 0.16), with larger P3 at the Cz (20.89 ± 0.83 µV) and Pz (19.62 ± 0.84 µV) than the Fz (18.57 ± 0.94 µV). The two-way interaction of group and site was not significant (*F*_2, 32_ = 0.92, *p* = 0.41, *η*^2^ = 0.06) (Figure 1).

#### 3.3.2. P3 Latency

**Figure 2:**
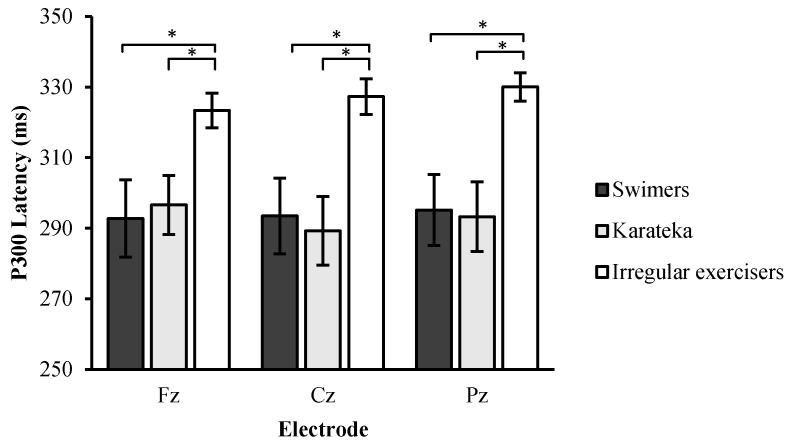
Mean P3 latency from the swimmers, karateka, and irregular exercisers during the auditory oddball task. * significant difference at *p* > 0.05.

Two-way ANOVA of group × site revealed a main effect of group (*F*_2, 32_ = 5.62, *p* < 0.01, *η*^2^ = 0.27), indicating shorter P3 latency in the swimmers (293.93 ± 34.84 ms) and karateka (293.08 ± 24.39 ms) than the irregular exercisers (326.89 ± 45.15 ms), while there was no significant difference between the swimmers and karateka (*p* > 0.05). The two-way interaction of group and site was not significant (*F*_2, 32_ = 1.15, *p* = 0.41, *η*^2^ = 0.06) (Figure 2).

### 3.4. The Relationship Between P3, RT, and Physical Activity

**Figure 3:**
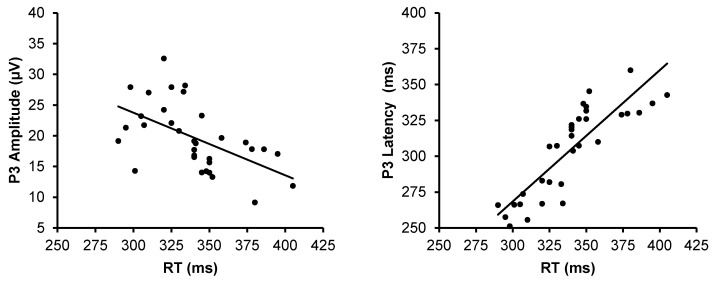
Scattergrams and correlation coefficients of P3 amplitude (left) and latency (right) from the averaged Fz, Cz, and Pz sites with the reaction time (RT).

**Figure 4:**
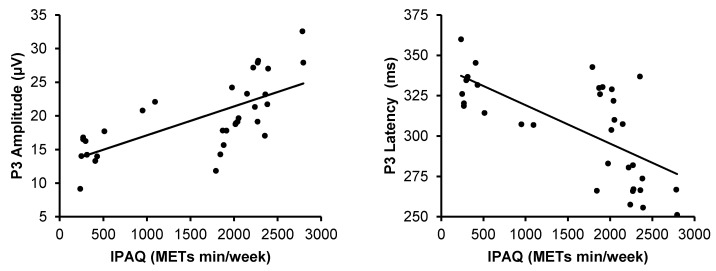
Scattergrams and correlation coefficients of P3 amplitude (left) and latency (right) from the averaged Fz, Cz, and Pz sites with the amount of physical activity calculated by the metabolic equivalent of task (METs min/week).

The correlation analysis was performed to examine whether RT was correlated with the P3 component averaged across midline sites (Fz, Cz, and Pz) among all participants (Figure 3). P3 amplitude and latency were significantly correlated with RT (*r* = −0.54, *p* < 0.01; *r* = −0.86, *p* < 0.01). Correlation analysis was performed to examine the relationship between physical activity and P3 component (Figure 4). The result showed that the amount of physical activity was significantly correlated positively with P3 amplitude (*r* = 0.69, *p* < 0.01), and negatively with P3 latency (*r* = −0.67, *p* < 0.01).

## 4. Discussion

The present study aimed to investigate the influence of sport experience with physical activity on attention, and to examine whether swimming and karate practices exert different effects on the neuroelectric system. The regular exercisers groups demonstrated significantly increased more extended exercise experience and physical activity relative to the irregular exercise group, which reflected the effectiveness of the group assignment process.

We hypothesized that regular exercisers (swimmers, karateka) would demonstrate an increase in attention ability and faster stimulus classification speed. Our results show that response time does not differ between groups. This result was inconsistent with Hillman, Castelli, & Buck (2005) with children using visual oddball task, and Pontifex et al. (2009) with younger adults using three-stimulus discrimination task. They reported that low-fit exhibited longer RT compare to high-fit [19,36]. The inconsistency may be driven by the diversity of task and age. In addition, the lack of significant difference in the RT in the current study may be due to the simplicity of the auditory oddball task, which requires low cognitive demands. Previous studies typically indicated more regular exercisers exhibit superior task performance relative to their less active counterparts during tasks that required higher cognitive demands such as task-switching task [37,38,39,40], flanker task [18,21,41], and Sternberg task [20], suggesting that the differences between regular and irregular exercisers could be observed with tasks that required higher-level of cognitive functions. 

Our results show that both regular exercisers groups exhibited larger P3 amplitude than the irregular exercisers. This finding is in agreement with electrophysiological studies that have documented significant differences of P3 amplitude between high-active/fit and low-active/fit preadolescent [18,36,40], indicating that exercise was related to greater attentional resources allocation during stimulus capture. Other researchers have replicated the physical activity–P3 amplitude relationship and extended it to include older adults using visual [42] and somatosensory [43] oddball tasks, flanker task [44], and task-switching task [37]. Regarding the sportsmen, ERP studies also suggested that habitual sport participation could promote neurocognitive development, such as inhibition control, as reflected by larger N2 amplitude [45]; and enhanced visual attention, as indicated by larger P3 amplitude [31]. 

Regarding P3 latency, our results also revealed that both regular exercisers exhibited shorter P3 latency compare to the irregular exercisers. This finding consistent with Hillman et al. (2006) study who used task switching paradigm with older (60–70 years) and younger (18–21 years) [40]. The study indicates that, regardless of age, physical activity was related to shorter P3 latency, suggesting that active lifestyle related to faster cognitive speed. This pattern of results was the first to suggest that regular exercise may affect cognitive processing speed with younger ages. However, it should be mentioned that not all studies have observed this finding [46], though methodological differences between the experimental paradigms may account for the disparate results. Other studies had examined the benefits of exercising with younger ages using cross-sectional design showing the potentially beneficial influence of exercising during preadolescent development [18,36,47,48]. Collectively, the available literature, with few exceptions, suggests a beneficial relation of being in a highly active lifestyle to neuroelectric indices of cognitive processing speed. In other words, the P3 latency data suggest that regular exercising decreases the time needed to capture information in the stimulus environment. Given this decrease in time for stimulus evaluation, it is likely that additional time is available for other cognitive operations occurring during decision making and response execution.

Functional MRI studies have continued examining exercise-induced changes in networks involving the prefrontal cortex and associated cognitive processes. Chaddock-Heyman et al. (2014) reported that higher-fit children had greater white matter integrity than lower-fit in several white matter tracts including sections of the corpus callosum, corona radiata, and superior longitudinal fasciculus [49]. Same research group observed greater gray matter volume in the hippocampus and basal ganglia in higher-fit children compared to lower-fit [50]. Schaeffer et al. (2014) showed that attendance in an 8-month exercise program was positively associated with improved white matter integrity [51]. Furthermore, Erickson et al. (2011) showed evidence of the positive effects of aerobic exercise on neuronal growth in the hippocampus size and improvement in memory function [52]. Additional evidence shows exercise increases the brain-derived neurotrophic factor (BDNF) [53,54], and white matter integrity [55]. Furthermore, a functional near-infrared spectroscopy (fNIRS) study suggests that acute physical exercise is associated with enhanced left dorsolateral prefrontal cortex activity during the Stroop task as well as improved performance in adults [56]. In summary, physical activity-related brain changes are associated with modulating the brain neural activity and leads to increase brain functioning of the executive system and better cognitive performance.

In the second hypothesis, we expected that karateka, as an open-skill sport, may exhibit better performance compared to swimmers as a closed-skill sport. The results indicated that there were no differences between karateka and swimmers, either in behavioral or neuroelectric levels. This finding was consistent with findings by Huang et al. (2014), who reported that there was no significant difference between closed- and open-sport exercisers in neuroelectric measures [35]. These two findings together suggest that sport experience, regardless of its type, is associated with general rather than specific cognitive enhancements. It should be noted that the lack of significance between karateka and swimmers in behavioral and neuroelectric levels may be due to the exercise duration was not enough to leave an impact, while with long-term training (i.e., after 15 years) the differences could be observed. Furthermore, oddball paradigm may not represent the neuroelectric differences between swimmers and karateka, and under high cognitive demands the differences may be found. The claim that different types of sports may elicit different changes in the neuroelectric system is much weaker because of the lack of statistical support. Therefore, further research is needed to fully characterize whether different types of sports can influence cognitive function through different mechanisms.

In the current study, the results indicate a significant relationship between RT and P3, which is in agreement with the work by Chueh et al. (2017), suggesting that shorter response time correlates with larger and/or shorter P3 [57]. Our findings also show that the P3 component significantly correlates with the amount of physical activity. This finding is consistent with previous work which focused on the influence of physical activity in ERPs [16], suggesting that physical activity might facilitate attention by the modulation of neural resource allocation to task-relevant stimuli. Several investigations using neuroimaging techniques have examined the relation of sport experience and physical activity to brain health and cognition. The underlying mechanism of the influence of these elements in neurocognitive performance remains under discussion.

## 5. Limitations

There are some limitations to the present study, which have to be considered. First, its cross-sectional design prevented causal inferences. Second, it’s a non-randomized trial that may lead to bias in the results. Third, auditory oddball paradigm was applied in the current study, which omitted the examination of other higher cognitive functions. Finally, we could not exclude all confounding factors that could bias the data such as physical fitness among all groups, and social context of the sports club with regular exercisers groups, which should be carefully considered in future studies.

## 6. Conclusions

Young adults who are involved with habitual sport participation exhibit more attentional resources and faster evaluation speed than those who are irregular exercisers and have an inactive lifestyle. Although karateka did not demonstrate improved performance compared to swimmers, the participants showed a numerically better neural efficiency during oddball task. Given that studies of physical activity effects on cognitive enhancements have typically focused on physical activity, the present study extends the previous findings by revealing that both swimming (as a closed-skill sport) and karate (as an open-skill sport) are relevant for improving the attention system in young adults. These results not only further our understanding of the relationship between different types of sports and cognitive benefits, but also open the door for further investigations that could examine the individuality of sports to reach an efficient prescription for cognitive benefits. This can be translated into real applications in physical and sports education programs, and can influence physical educators, coaches, and people who are responsible for public health to promote specific sports or activities based on peoples’ individuality. 

## Figures and Tables

**Table 1:** Descriptive data for participants’ demographic and physical characteristics among three groups (mean ± SD).

Variables	Swimmers (*n* = 11)	Karateka (*n* = 11)	Irregular Exercisers (*n* = 11)
Age (yrs)	19.64 ± 1.43	19.45 ± 1.04	20.09 ± 1.30
Education (yrs)	13.55 ± 1.21	13.45 ± 0.93	13.91 ± 1.22
Height	1.78 ± 0.05	1.75 ± 0.07	1.73 ± 0.04
Weight	74.90 ± 6.64	73.02 ± 7.37	74.36 ± 4.50
BMI (kg/m^2^)	23.74 ± 1.45	23.69 ± 1.12	24.87 ± 1.55
*Sport experience*			
Training (years)	5.44 ± 1.63	6.74 ± 0.85	0.50 ± 0.25 *
Number of sessions (week)	3.18 ± 1.07	3.45 ± 1.29	0.00 ± 0.00 *
Time (min)	90.00 ± 23.23	73.63 ± 20.62	0.00 ± 0.00 *
*Physical activity (IPAQ)*			
Vigorous (MET)	500.91 ± 111.00	429.27 ± 127.30	38.18± 75.74 *
Moderate (MET)	786.64 ± 190.85	852.36 ± 132.49	219.18 ± 105.94 *
light (MET)	917.91 ± 140.96	866.55 ± 270.47	199.00 ± 79.42 *
Total MET-min/week	2,205.45 ± 213.21	2,148.18 ± 248.47	456.36 ± 294.02 *

BMI, body mass index; IPAQ, International Physical Activity Questionnaire; MET, metabolic equivalent of task. * represents the significant difference between the groups, *p* < 0.05.
